# Health Policy in Japan – Current Situation and Future Challenges

**DOI:** 10.31662/jmaj.2018-0016

**Published:** 2019-03-04

**Authors:** Shinya Matsuda

**Affiliations:** 1 Department of Preventive Medicine and Community Health, School of Medicine, the University of Occupational and Environmental Health, Kitakyushu, Japan

**Keywords:** universal coverage, Diagnosis Procedure Combination, claims data, health policy

## Abstract

Demographics and disease structures have a great influence on medical service delivery systems and their finances. Japan has a rapidly aging population, with those aged 65 or over accounting for 27.4% of the population. Total Fertility Rate was 1.44 in 2016 and these combined factors, fertility rate and aging population, have seen the total population fall since 2006. Consequently, there is an increase in users of social and health services and a decrease in tax payers. This requires the Japanese government to reorganize its social security system.

In order to reorganize the health service delivery system, the Ministry of Health, Labor and Welfare (MHLW) has started to collect data in the form of Diagnosis Procedure Combination data and the National Receipt Database. The former gathers around 11 million discharged cases from around 3,000 acute care hospitals annually. The latter gathers more than 1.7 billion claims data from all medical facilities each year. Using these methods, the Japanese government is trying to proceed with a data-driven health reform. As the principle of the Japanese healthcare system is a private dominant supply system under public financing, the existence of appropriate information regarding health needs is crucial for reliable administration.

The success of this policy depends on strong leadership by politicians with a clear direction for the future. Additionally, it is necessary to improve the ability to utilize information in society as a whole. The author believes that strengthening the foundations of health service research is crucially important for public health administration in Japan.

## 1. Introduction

Since the establishment of the universal health insurance scheme in 1961, Japanese citizens have enjoyed a generous system under which the insured members have freedom to access health care facilities and a wide range of medical services for a relatively low co-payment. Although Japan’s health system is categorized as a social insurance system, it receives a considerable amount of tax subsidy.

Since the attainment of universal coverage in 1961, the government has made continuous efforts to equalize the benefits between occupational and community settings. Previously, out of pocket payment (OPP) levels were vastly different between the two settings. In the case of the insured, the former was a nominal amount and the latter was 50%. In order to equalize the OPP level, the government introduced subsidies for financially weak insurers. For community-based insurers, subsidies accounted for about 50% of total revenue. However, over the course of the two decades of economic stagnation which started in the 1990s, subsidies for health insurance became a heavy burden for the government and for occupational setting insurers. Indeed, Japanese financial policy during this period was heavily dependent on deficit bonds, which resulted in a total of US$10.6 trillion of debt as of 2017 (1USD = 113JPY) ^(1)^. Large parts of this debt were caused by governmental subsidization of social insurance. For this reason, control of social insurance expenditures has become an important political issue.

As the Japanese social insurance system depends on the transfer of money from the working generation to the retired, the increase in the aged population and the decrease in the working population have caused financial difficulty. Additionally, the aging population has led to a change in disease structure, wherein multiple co-morbidities and chronic conditions are more common in patients. These changes require structural reform of the healthcare system.

Another important issue requiring attention is the increasing awareness of quality of care among Japanese citizens. Since the year 2000, medical accidents in university hospitals and other major hospitals have occurred one after the other, raising substantial public interest in the quality of medical care. One consequence of this is that hospitals have been asked to disclose more information about medical safety and the quality of medical care.

In this article, the author will explain the Japanese healthcare system and the recent reform of health policy in view of the above-mentioned situation.

## 2. Background Information Relevant to Healthcare Reform in Japan

### (1) The Japanese administration system and financial situation

**Figure 1.**
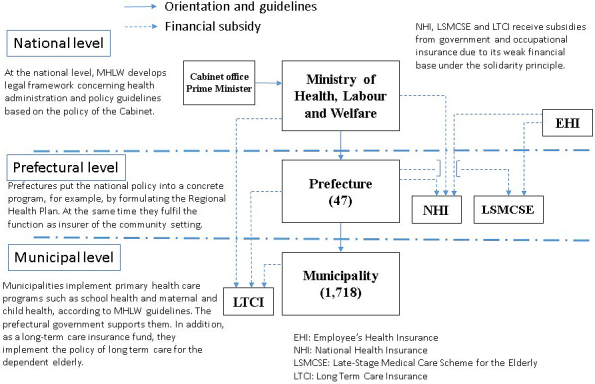
Implementation of health policy in Japan.

The Japanese administration system comprises three levels: central government; 47 prefectures; and 1,718 municipalities. The Ministry of Health, Labor and Welfare (MHLW) establishes the principles and basic laws. The national health policy is implemented through local governments, as is shown in Figure 1. Most basic health and welfare services, such as those for mother and child and the elderly, are provided by municipal government. The prefecture coordinates activities among the municipalities.

The declining population in Japan has worsened the finances of small local governments. As a result, it has become difficult for municipalities to operate the community-based insurance scheme and since 2018 the prefectural governments have assumed this financial responsibility.

Japan tends to hold general elections more frequently than other countries. Indeed, as many as 15 prime ministers held power between 1990 and 2018. The shortest term was a serving of only two months. The main reasons for this frequent replacement have been cabinet scandals, which have included prime ministers, and power struggles within political parties. Media coverage of these situations has caused distrust of politicians to grow. One result is that it is now difficult for the government to establish long-term programs for the aging society. The unwavering support of populism by politicians has resulted in unnecessary public investment and postponement of crucial reforms. Furthermore, there are instances where political rivalry has worsened the situation, for example, by the duplication of similar programs.

Japan’s primary balance has been negatively affected because the government has repeatedly supplemented revenue shortages by issuing government bonds. The postponement policy was acceptable when the economy was expanding, but might cause major financial problems when the economy is shrinking and the labor force is decreasing. Today, a quarter of the total national budget is spent on the payment of national bonds, the worst ratio being among the Organization for Economic Cooperation and Development (OECD) countries.

### (2) Demographics

According to official statistics on 1 October 2017, the total population of Japan was 127 million, of which 48.7% were male and 51.3% were female. Since 2010, the total population has decreased by 1.35 million (−1.06%) ^(2)^.

Table 1 shows the changes in the population according to age and gender since 1980 ^(2)^. As seen in the table, the Japanese population is rapidly aging. According to estimates by the National Institute of Population and Social Security Research, 30.5% of the population will be over 65 years old in 2025 ^(3)^. The most important factor associated with these changes is a decrease in total fertility rate, which has dropped from 1.75 in 1990 to 1.43 in 2017. Principal factors behind this phenomenon include an increase in the number of people not getting married, a higher age at marriage, and an older maternal age at the birth of the first child.

**Table 1.** Chronological Changes of Population Indicators, Selected Years.

Indicators	1980	1990	2000	2010	2016
Population(in thousands)	117,060	123,611	126,926	128,057	126,940
Female (% of total)	50.8	50.9	51.1	51.3	51.4
Population (% of total)					
0–14 years	23.5	18.2	14.6	13.1	12.4
15–64 years	67.4	69.7	68.1	64.1	60.3
65 years and older	9.1	12.1	17.3	22.8	27.3
Mean age at first marriage					
Male	27.8	28.4	28.8	30.5	31.1
Female	25.2	25.9	27.0	28.8	29.4
Mean age at first child (mother)	26.4	27.0	28.0	29.9	30.7
Total Fertility Rate	1.75	1.54	1.36	1.39	1.44

Sources: Statistics Bureau Ministry of Internal Affairs and Communication, 2017

An aging society means that the number of elderly households is also increasing. An elderly household is a private household with members aged 65 or over. The percentage of single-occupancy households increased from 8.6% in 1975 to 27.1% in 2015, while the percentage of single-occupancy households with elderly couples increased from 13.1% to 31.1% over the same time period ^(4)^. This situation requires the socialization of care for the elderly. The MHLW introduced the Long-Term Care Insurance (LTCI) scheme in 2000 to solve this problem ^(5)^.

### (3) Health statistics

Since World War II, Japan’s health status has dramatically improved, as is seen in Table 2 ^(6)^. Today, Japan is ranked one of the healthiest countries in the world. Contributory factors include improvements in general hygiene, medical systems and nutrition, a lower incidence of violent and fatal accidents, and a moderate climate. Furthermore, there is no doubt that the universal health insurance system that was established in 1961 has played an important role in the improvement of health.

**Table 2.** Chronological Changes of Health Indicators, Selected Years.

Indicators	1970	1990	2000	2010	2016
Age adjusted mortality rate (per 1,000 population)					
Male	12.3	7.5	6.3	5.4	4.9^*^
Female	8.2	4.2	3.2	2.7	2.5^*^
Death rate of top 4 causes (per 100,000 population)					
Malignancy	116.3	177.2	235.2	279.7	298.2
Cerebro-vascular diseases	175.8	99.4	105.5	97.7	87.4
Heart diseases	86.7	134.8	116.8	149.8	158.2
Pneumonia	27.1	55.6	69.2	94.1	95.3
Neonatal mortality rate (per 1,000 live births)	8.7	2.5	1.8	1.1	0.9
Infant mortaloty rate (per 1,000 live births)	13.1	4.6	3.3	2.4	2.0
Maternal mortality rate (per 100,000 births)	48.7	14.0	10.0	6.0	5.0
Life expectance at birth
Male	69.3	75.9	77.7	79.6	79.9
Female	74.7	81.8	84.6	86.3	86.3

^*^: 2015 Sources: Statistics Bureau Ministry of Internal Affairs and Communication, 2017

Alongside economic development, disease patterns in Japan have also changed, from predominantly acute to chronic diseases. Today the three major causes of death (cancer, heart diseases, and cerebrovascular diseases) account for 60% of all deaths.

In 2008, a health checkup and health education system were introduced for people between the ages of 40 and 75. These measures also help to prevent metabolic syndrome, which is a risk factor for certain chronic diseases.

## 3. Health Systems

### (1) Health insurance system

**Figure 2.**
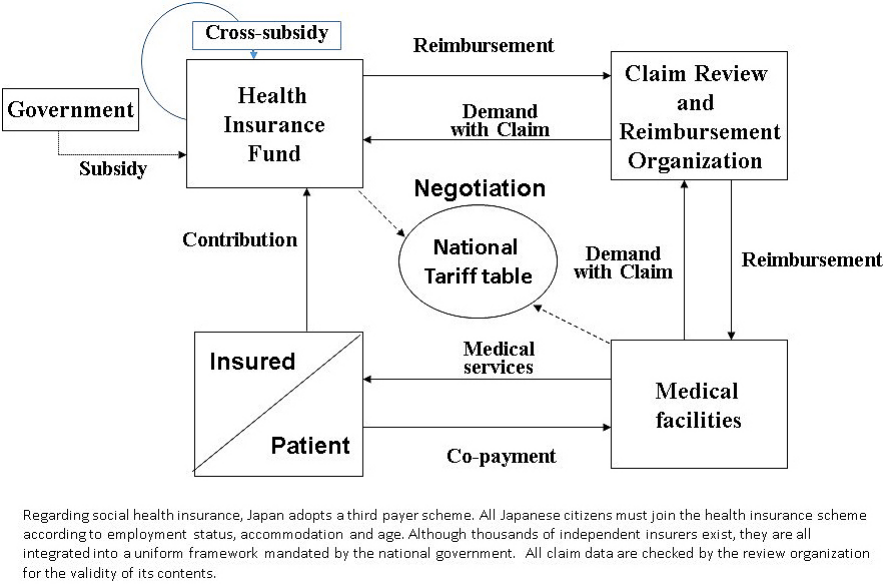
The structure of the social medical insurance scheme in Japan.

Figure 2 explains the Japanese public insurance scheme. All Japanese citizens must join the health insurance scheme according to employment status, accommodation, and age. Although thousands of independent insurers exist, they are all integrated into a uniform framework that is mandated by the national government. The Japanese health system is based on fee-for-service reimbursement under a uniform national tariff schedule. The health insurance scheme is categorized into three basic groups according to age and employment status: the Employees’ Health Insurance scheme (EHI) for employees and their dependents; the National Health Insurance scheme (NHI) for the self-employed, farmers, the retired and their dependents; and the Late-Stage Medical Care Scheme for the Elderly.

The insured pay the premium of their own insurance. In the case of EHI, the average rate is 10% of a salary with a cap of 13%, of which one half in principle is paid by their employer. For the NHI, the local government determines the premium rate. The calculation formula and amount of contribution differs in accordance with the local governments from an average of US$2,586 per year to US$5,635 per year. For NHI, as its financial base is weak, about 50% of the total cost is covered by governmental subsidies and cross-subsidies. In the case of the Late-Stage Medical Care Scheme for the Elderly, the insured contributes 10% of the total cost, 50% is financed by government subsidies, and 40% is subsidised by the contribution of the working generation. In 2015, 58.7% of the total population was covered by EHI, 28.3% by NHI, and 12.4% by the late-stage scheme ^(7)^.

When the insured receive health services at a medical facility, they are required to pay 30% of the total cost as an OPP. Those aged 75 or older and children under 6 years of age pay 10%, while those aged 70-74 pay 20%. The threshold of monthly co-payment is US$707 for those aged 50 years with annual incomes of US$50,000. This cap differs according to annual income, age, and frequency of medical service use.

As Japan uses the third-party payer system, the medical facilities ask for reimbursement of the balance of the charge for patients through the Claims Review and Reimbursement Organizations (CRROs), established in all 47 prefectures. The expert committees of CRROs review all the claims submitted from the medical facilities to the office for appropriateness of procedures. In the case of inappropriate or unnecessary procedures, CRROs can deny the reimbursement.

### (2) Tariff schedule

In Japan, almost all medical facilities are paid by the social health insurance scheme. The basis of reimbursement is the national tariff schedule. In principle, this tariff schedule is revised every two years after negotiation at the Central Social Medical Insurance Council (CSMIC). This council is comprised of representatives of payers’ organizations, providers’ organizations, and those representing the public interest from the MHLW.

There are two steps for fee schedule revision. At first, the prime minister’s cabinet sets the modification rate of total medical expenditures. After determining the share of each medical sector (e.g., physicians, dentists, pharmacists), the MHLW modifies the fees for each procedure according to the result of the Survey on Economic Conditions in Health Care and according to negotiation at CSMIC. As Ikegami et al. indicated in their study, the national tariff system has two objectives ^(8)^. Firstly, it controls the total expenditures. Secondly, it sets fees for particular procedures in a way which incentivizes physicians to modify their behavior. For example, the current tariff schedule includes an incentive to provide more home-based care. This indirect but fine-tuned method of cost control is a useful tool for the MHLW in changing health policy.

### (3) Health resources and service delivery system

On 30 March 2018 in Japan, there were 8,389 hospitals, 101,860 clinics, and 68,756 dental clinics. More than 80% were privately owned. In comparison with other OECD countries, a higher density of hospital beds per capita and longer length of stay characterized Japan (Table 3) ^(9)^. In 2014, the number of physicians, nurses, and pharmacists was around 2.45, 11.61, and 2.27 per thousand inhabitants, respectively (Table 4). Japan also has a relatively small number of physicians and high number of pharmacists in comparison with France, Germany, the UK, and the USA.

**Table 3.** Health Care Workers per 1000 Population in Different OECD Countries.

Indicators	Japan^*^	USA^**^	France^**^	Germany^**^	UK^**^
Practicing Physicians	2.4	2.6	3.3	4.1	2.8
Practicing Dentists	0.8		0.6	0.9	0.5
Practicing Pharmacists	1.7		1.1	0.6	0.8
Practicing Nurses	11.0	11.3	9.9	13.3	7.9

^*^: 2014, ^**^: 2015 Sources: OECD Heath Statistics 2017

**Table 4.** Hospital Care Output in Different OECD Countries.

Indicators	Japan	USA	France	Germany	UK
Hospital beds per 1000 population	13.2^*^	2.8^**^	6.1^*^	8.1^*^	2.6^***^
Average length of stay (curative care ward)	16.5	6.1	10.1	9.0	7.0

^*^: 2015, ^**^: 2014, ^***^: 2016 Sources: OECD Heath Statistics 2017

In Japan, doctors can freely choose where to set up a clinic and patients are allowed unrestricted access. In contrast, the establishment and expansion of hospital beds has been strictly regulated under the Health Care Law since 1985. Nevertheless, hospitals are allowed to purchase any equipment and to open any specialty department.

Although these characteristics are considered to be associated with high costs, Japan has been successful in controlling costs when compared with other OECD countries. Hashimoto et al. in their study suggested that Japan’s outpatient-focused healthcare system is one of the main reasons for this ^(10)^. In fact, according to the OECD Health Statistics from 2016, the number of outpatient service visits per capita in Japan was 12.8 (2013). This figure is higher than the equivalent figures of Germany (9.9 in 2014), France (6.3 in 2014), and the USA (4.0 in 2010) ^(9)^.

One of the most important problems of health resource utilization in Japan is its unequal distribution among geographical regions and sub-specialties. The Japanese government has implemented various measures to solve the geographical disparity of health professionals, for example, by increasing the number of students studying medicine and by introducing a system whereby high school students in rural areas can preferentially enter medical school. More time is required to evaluate the full effects of these policies.

Another problem is a lack of indicators for physician manpower requirements by specialty. Medical students in Japan are free to choose their specialty after graduation. This has contributed to an uneven distribution of physicians by specialty and subsequent mismatch between supply and demand in certain specialist areas. The MHLW committee has discussed the problem of uneven distribution but is yet to find an effective solution. Some members of the committee have suggested that there should be restrictions on the geographical arrangement of medical professionals, such as that occurring in France and Germany.

### (4) Medical expenditure

National health expenditures in Japan are increasing. In 2014, expenditure totaled US$361.1 billion, equivalent to US$2,842 per person ^(11)^. This figure corresponded to 11.2% of the National Income. According to the OECD database, the total healthcare expenditure in Japan was 10.9% of the GDP, which was 2% above the median for OECD countries. Expenditure, according to type of medical service, was as follows: inpatient hospital care (37.4%); outpatient care (34.3%); pharmacies (17.9%); dental clinics (6.8%); meal services for inpatients (2.0%); and nursing care home visits for the elderly (0.3%).

In 2014, the cost of healthcare for the elderly reached US$1,085 billion. This figure is rapidly increasing and now accounts for as much as 35.4% of the total national healthcare expenditure.

A structural analysis of the increased rates of national healthcare expenditure between 2014 and 2015 revealed that within the total growth rate of 3.8%, there was a reduction of 0.1% in rate of national healthcare expenditure due to a population decrease, an increase of 1.2% because of aging (change in the population structure by age cohort), and a natural increase of 2.7% in rate of national healthcare expenditure, which included the effects of advancing medical technology ^(12)^. This result highlights the considerable impact of aging and advancing medical technology on increasing healthcare costs.

## 4. Challenges for Future Health System Reform in Japan

Due to the drastic changes in demographics and disease structures, Japan needs to reconstruct its social system to make it sustainable. In this section, the author will discuss some current healthcare reform programs and challenges.

### (1) Improving public awareness of the social security system

The number of citizens who do not pay the premium for their pension is increasing, especially among the younger generation. In 2015, less than 60% of 30 to 39 year olds paid their premiums ^(13)^. One reason for this is that many people believed a private pension was more rewarding than the national pension scheme. This is a fundamental problem in the sustainability of the Japanese system, for which solidarity is a base principle. There is criticism that the Japanese public do not have sufficient understanding of the purpose and structure of social security schemes due to a lack of education on this topic. In responding to this criticism, the MHLW has prepared teaching materials to promote and educate junior high and high school students with regard to social security ^(14)^. Unfortunately, these resources have not yet been fully utilized. It is necessary to increase public awareness and understanding of these social security systems.

### (2) Increasing transparency for better choices and quality improvement

To facilitate reform, the Japanese government has implemented a standardized infrastructure for health information, using the Diagnosis Procedure Combination (DPC) database and National Receipt Database (NDB). With these databases, the MHLW is trying to establish a data-driven negotiation system to replace the current corporatist approach.

Currently, all digitized claims data are gathered by the MHLW and are registered at the NDB ^(15)^. This database is constructed on an individual insured basis. The ID number is treated by the Hash function conversion twice in order to make it anonymous, while still allowing the combination of all data for individuals. The database contains detailed information such as diagnoses, age, sex, provided procedures, and used materials (i.e., drugs and devices) with information including date, volume, and tariff. More than 1.7 billion records are registered into the NDB annually. Since 2011, the MHLW has started to provide this data to researchers and local governments to facilitate their health service research and to create data-driven health policies.

**Figure 3.**
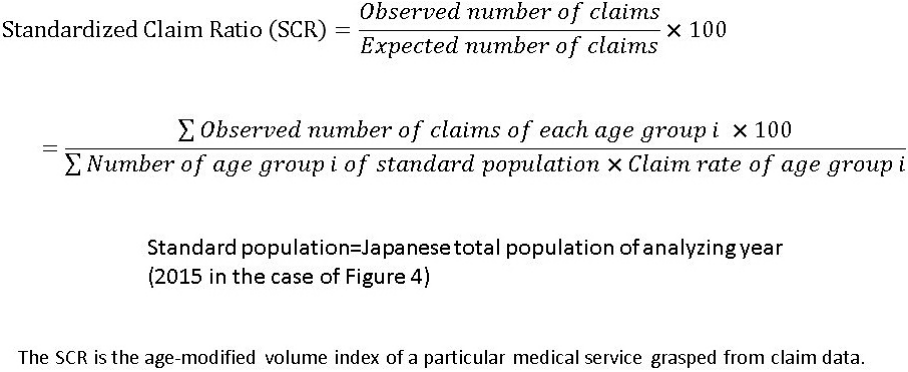
Formula of Standardized Claims Ratios.

One example of information derived from the NDB is the age-modified volume index of a particular activity, known as the Standardized Claim Ratio (SCR). The formula for calculating SCRs is shown in Figure 3. The current status of service delivery can be analyzed using SCRs. For example, if one region shows SCRs of below 80 for outpatient consultations, 90 for acute inpatient care, 120 for chronic inpatient care, and 40 for home-based care, the region may have a problem in primary care. Further evaluation may reveal the cause of the problem; for example, a shortage of primary care physicians leading to difficulty in expanding home-based care. In this case it would be practical to maintain the chronic inpatient care instead of increasing the volume of home-based care, even though the MHLW recommends replacing chronic inpatient wards with home-based care. On the contrary, the Tokyo metropolitan area shows a higher SCR for acute inpatient care and outpatient consultations, but a very low SCR for chronic inpatient care. Within the coming few years the elderly population of the Tokyo metropolitan area will substantially increase. Considering the difficulty in expanding inpatient chronic care in Tokyo, the only solution would be to expand home-based care. As in these examples, local policy makers would be able to discuss future health policy based on SCR analysis ^(16)^.

**Figure 4.**
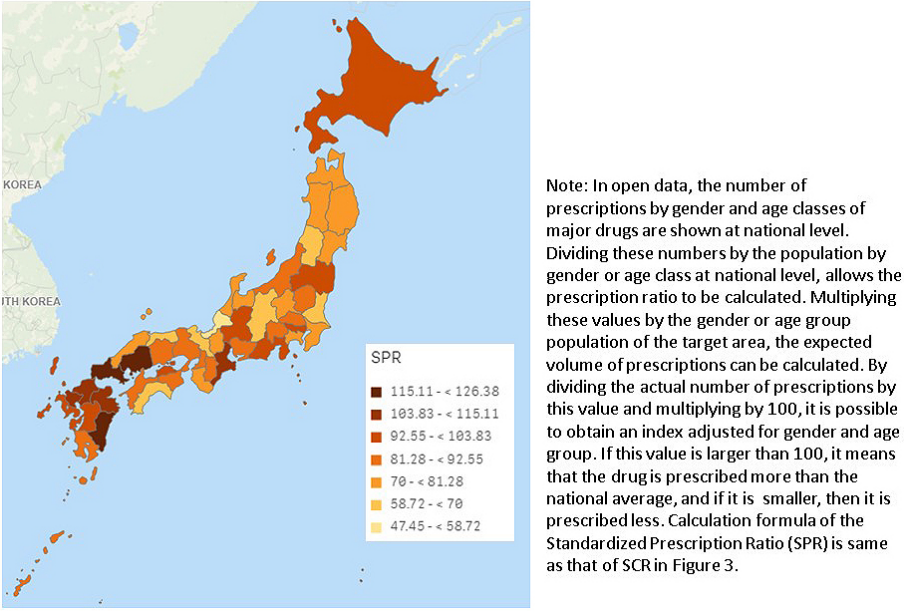
Geographical variation in levofloxacin use (2015).

In addition, the MHLW provides the open data from the NDB in the form of summary tables for procedures and drugs ^(17)^. Although this open data has limited content, it is still possible to conduct analyses useful for policy making. Figure 4 shows an example of our research on pharmaceutical use. There is wide geographical variation in levofloxacin use. These findings will be useful for objective discussion, as appropriate use of pharmaceuticals is one of the most important issues of health policy, both for quality of care and for the health economy.

Alongside the NDB, the MHLW gathers casemix data from approximately 3,000 acute care hospitals ^(18)^^, ^^(19)^. Since 2005, most acute care hospitals have been paid by the DPC. In 2017, the DPC scheme covered 550,000 beds and 11 million discharged cases. The DPC tariff is a flat-rate per day, which changes depending on the length of stay. The hospital is required to submit a discharge summary and detailed data for all procedures provided during hospitalization as digitized data. One of the biggest advantages of the DPC system is that hospital activities become more transparent. The MHLW discloses the discharge volume data from each DPC hospital every year. This allows the public to view the health service delivery status in each region, including which hospitals treat the most cancer cases, functional differentiation between hospitals, and chronological changes in the performance of each hospital (volume and length of stay).

Another important contribution of the DPC system is that it facilitates the systematic evaluation of hospital care quality through the development of quality indicators (QI). As DPC gathers very detailed process data, it is possible to formulate a series of QIs. Many hospital groups, such as the National Hospital Organization and Saiseikai, publish their annual QI report on their websites ^(20)^^, ^^(21)^. It is expected that these projects would use more sophisticated mechanisms in other countries, such as NHS Choice in the UK and Hospital Compare in the USA.

### (3) Reorganization of medical education and post-graduate training

The current changes in disease structure require more general practitioners who can treat elderly patients with multiple chronic co-morbidities. Currently, Japan is trying to introduce a subspecialty board system without considering this fact. As noted in the study by Shibuya et al., the lack of quotas has led to the training of too many specialists ^(22)^. Only a small proportion continue in their chosen specialty, and the rest become “generalists” without formal training as general practitioners. Shibuya et al. in their study suggest that Japan should conduct a long-range reform of medical education, including more organized undergraduate education in general practice, as well as a retraining program in general practice for subspecialty physicians. In fact, since 2016, the Japan Medical Association has organized a continuous training course for family doctors ^(23)^.

Demand for general practitioners is increasing in many developed countries due to rapidly aging populations and the increase in patient stakeholders with chronic diseases. In France, general practitioners are defined as specialists, and a mechanism has been introduced to determine and foster the necessary number of practitioners by each region. In the United Kingdom and the United States, a sophisticated training program for general practitioners has long been established. It will be necessary for Japan to re-examine these general practitioner training systems with reference to the experiences of such countries.

### (4) Sharing the burden among stakeholders

Coping with fundamental structural change requires Japan to reconstruct the financial system for social insurance or its service delivery system. These changes led to the government passing the Healthcare Structural Reform Package Act in 2006. It is important to recognize that this Act treats the reform of the service delivery system and the insurance system as one package for the first time. Establishment of the Securing Medical Care for the Elderly Act, has transferred the responsibility of medical care from municipal to prefectural governments for the elderly, and has obliged the latter to monitor and control related health expenditures. In 2008, the Late-stage Medical Care System for the Elderly was introduced. In addition, each prefectural government is required to establish a Prefectural Plan for the Optimization of Health Expenditures and for a more practical Regional Health Plan (RHP). Together, these might facilitate the functional differentiation of health service providers.

In comparison with similar systems in other countries, these plans in Japan are less efficient in promoting structural change of the service delivery system. One major reason is that the Japanese plan regulates the number of beds but does not regulate more advanced medical technology, which might have a greater effect on service quality and cost optimization. For example, in France there are strict regulations concerning the number of cases and staffing with respect to advanced medical treatments such as cardiac surgery and proton beam therapy. The French RHP, “le schéma régional d'organisation sanitaire (SROS)”, is a political tool to geographically optimize medical service delivery taking these conditions into consideration. Furthermore, the SROS establishes the number of services, collaborations with other facilities, and also the quality index of medical care to be achieved by each medical institution. These medical institutions are required to contract with local authorities in order to provide care. It is necessary to reconsider the method of improving the effectiveness of Japan’s RHPs with reference to the SROS in France.

Due to the fundamental changes in socio-economic conditions, Japan is required to implement cost-containment policies for the health sector. However, it is natural that as the elderly population increases, social security expenses also rise. It must be recognized that the most important role of health policy is to provide equally high quality medical care to all citizens. Article 25 of the Constitution clarifies the role of government in social security and health policy. It states that “all people shall have the right to maintain the minimum standards of wholesome and cultured living” and “in all spheres of life, the State shall use its endeavors for the promotion and extension of social welfare and security, and of public health” ^(24)^.

The problem is that the financial resources required to cover these costs have not been secured. There are two ways of remedying this situation. Firstly, is to reduce the cost of supplies, either by a restriction of coverage or by reduction in tariff. Secondly, is to increase financial resources by increasing taxation, or by raising premiums. France and Germany regularly review the price of pharmaceuticals covered by public insurance. Decisions are made to try and optimize expenses from the viewpoints of efficacy and whether the drug is provided as an over-the-counter medication. It is necessary to re-evaluate the range of pharmaceuticals covered by Japanese public health insurance.

An increase in the consumption tax of 1% would equate to an extra US$0.88 billion in tax income. The current ruling political party, the Liberal and Democratic Party (LDP), previously tried to increase the consumption tax from 5% to 10% in 2015. However, they raised it to 8% in 2014 and postponed the remaining increase until 2019, because of fear of causing a downturn in the economy and losing the election. The author considers that the increase in consumption tax is indispensable to securing of finance for an improved social security system. It must be noted that the consumption tax rate in Japan is lower than in many other developed countries, including France (19.6%) and Germany (19.0%).

In addition to raising the consumption tax, it is also necessary to evaluate the range of income from which premiums are calculated. In 1991, the French model for calculating income subject to calculation of premiums changed from labor income to total income, including assets income. Such methods would likely be reasonable in terms of securing economic vertical equity in Japan, where the social insurance system is based on the social solidarity principle.

Another way to increase financial resources is to increase the size of the labor force. To do that, it is necessary to make Japanese society an ageless society. In addition to reviewing working conditions and the retirement system, the role of medical care in supporting people to be active is also important. In the UK, doctors have a resource called the fit note ^(25)^. This enables general practitioners to issue medical certificates to employers describing the considerations necessary for patients with illnesses to be able to adapt to the workplace. This could be useful in Japan for elderly employees with medical issues. Furthermore, given the worsening situation in the Japanese labor market, it is likely that more foreign workers will be accepted in Japan. This will require a review of the qualifications of the insured and dependent persons. Failure to properly respond to this problem could result in confusion over immigrants, similar to that seen in Europe.

Regardless of what measures are taken, it is inevitable that structural reforms mean that the additional burden will be shared among stakeholders. For proper policy selection, an arrangement is necessary to arrive at a reasonable consensus among stakeholders based on objective data. Comprehensive infrastructure for medical information is required for this purpose.

## 5. Conclusion

Due to an aging population, advances in medical technology, changes in the economic environment, and increasing awareness about the quality of care, medical system reform is indispensable. However, because of broad disappointment with the regime change of the LDP to the Democratic Party of Japan in 2009, Japanese citizens feel more conservative toward change. Given this social atmosphere, it would be practical for the reform strategy to be incremental, even though it requires time. In order to make this policy successful, Japan needs long overdue strong leadership from politicians. It is also necessary to improve the ability to utilize healthcare information in society as a whole. Finally, the author believes that strengthening the foundation of the Health Service Research is of critical importance for public health administration in Japan.

## Article Information

### Conflicts of Interest

None

### Author Contributions

This article was completed by the author alone.
